# Supplementary material for: Tweezepy: A Python package for calibrating forces in single-molecule video-tracking experiments
Source: PLoS One. 2021 Dec 31;16(12):e0262028. doi: 10.1371/journal.pone.0262028 (PMC8719779; doi:10.1371/journal.pone.0262028)
Supplement: Attachment — Submitted filename: tweezepy_reply.pdf [file pone.0262028.s001.pdf]

Reply to reviewer comments for Tweezepy

Ian L. Morgan

October 28, 2021

Reviewer one

We appreciate the reviewer's kind comments that the "article is overall well written and the topic well explained." As the reviewer notes, while the topic has been extensively described in the past, an automated program is of "interesting use in general in the field." We agree with the reviewer that an "interesting feature and strength of this program is that it offers different robust methods to be able to fit as accurately as possible their data and also provides the users with automated robust methods to estimate biases and errors of their fit."

The following are responses to the reviewer's suggested changes:

> *In the introduction the authors discuss forces and calibration, could they provide a short description relating this calibration to an actual molecule as an example where they show forces and the parameters they want to fit (γ and κ) in for example a small illustration?*

We have added an introductory figure (Fig. 1) that shows the basic geometry, including the forces and parameters of interest. We also modified the text in the background section (lines 77-79 and 89-91) to provide a short descriptive example of calibrating the force on an actual molecule in an SMFS experiment.

> *Can they explain which molecules are better to be used experimentally for calibration purposes using their methods?*
> *Can they discuss for which force regimes it is better to use PSD or Allan variance?*

We have modified the text to include a short discussion on this topic (lines 118-124). As described in the modified text, conditions with intermediate to larger relaxation timescales, $\tau_c \equiv \gamma/\kappa$, with respect to the sampling time will give more accurate parameter estimates. In general, more flexible and longer polymers, as well as smaller forces will have larger relaxation timescales.

> *In line 113- 118, the authors mention complementarity of PSD and Allan variance, can they explain how to combine them in terms of force calibration?*

We have modified the text to clarify our statement and answer the reviewer's question (lines 129-133). When working with a new instrument or modified instrument, we suggest analyzing bead trajectories with both the PSD and AV. The PSD will help to identify high frequency coherent noise sources, and the AV will help identify low frequency coherent noise sources. Once these noise sources are identified and accounted for, either the PSD or AV can be used to calibrate forces.

> *In eq 4, the authors should mention that $P_A(f)$ is the experimentally measured Power spectrum.*

Eq. 4 in the main text refers to the expected or analytical PSD values that accounts for the exposure time of the camera. The experimental PSD values are calculated in Eq. 11. We have modified the text to clarify this point (lines 152-153).

> *In line 202: is there a difference in terms of results for the PSD using Welch's or the FFT Cooley-Tukey algorithm?*

Welch's method is the process of binning and windowing, and then averaging multiple independent power spectra to reduce the noise in each PSD estimate. Each of the independent power spectra are calculated using the Cooley-Tukey algorithm. Hence, they are not alternative methods for calculating the power spectrum. We have adjusted the text to clarify this point (lines 207-209).

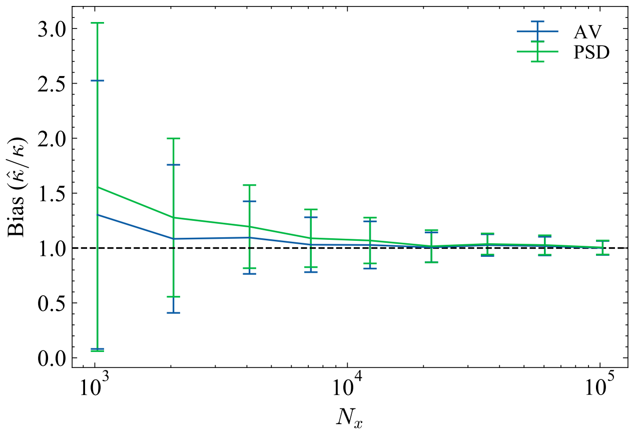

Figure 1: Bias scales with the number of points, N_x, in the simulated bead trajectory. Parameters were estimated using the AV method (blue) or PSD method with three half-overlapping bins (green). Simulations were carried out as described in the main text with parameters $f_s = 100\,\text{Hz}$, $\gamma = 1.00 \times 10^{-5}\,\text{pNs/nm}$, and $\kappa = 6.28 \times 10^{-6}\,\text{pN/nm}$. Errorbars represent one standard deviation.

Are the simulated traces mentioned in section 6 and 7 the same than in fig 1 and 2? If yes please refer it in text.

They are indeed the same as in Figs. 1 and 2. We have adjusted the captions accordingly.

Line 341: can authors explicit what they mean by "previously estimated noise type" is it from previous points in AV?

When there are ten AV points, the Greenhall algorithm can typically differentiate between the diffusive and thermal regimes of the bead for the first seven points but not the last three points. This is important for determining the equivalent degrees of freedom. To circumvent this problem, we use the type of noise, e.g., thermal, of the seventh point to determine the equivalent degrees of freedom for the last three points. We have adjusted the text to clarify this process (lines 356-359).

In line 410: authors say "this bias can be reduced by increasing the length of the trajectory" can authors show this improvement in their figures? Why are only examples of short traces shown?

We sought to compare our results to previous computational implementations of the AV and PSD methods in Ref. [1] to demonstrate Tweezepy's accuracy. In both Ref. [1] and our results, there is an increase in the bias at low corner frequencies. MLE is known to have a small bias that scales as $1/n$ [2], where n is the number of sampled relaxation times. Indeed, when the corner frequency is kept constant, increasing the length of the trajectory, and thus the number of sampled relaxation times, reduces the bias in the κ estimate (Fig. 1).

Line 411 onward: there is an unclear description of the minimum in bias and error in relation to figure. As it is supposed to be the case for both parameters, why are only C and D mentioned? There also seem to be no clear minima in the bias for γ as well as its error for $f_c > f_s/8$ which looks more like an increasing tendency. Is this observation only valid for κ? In this case it is not very consistent with the rest of the description.

We have revised the text to clarify this point (lines 437-439). In agreement with previous results [3, 1], both the error in κ and γ both increase when $f_c > f_s/8$. This results in a minimum in the error in κ but not γ. In our results, we find that fixing γ eliminates the increase in κ error, and thus, the minimum.

In general the figures and their legends are also not very clear and I suggest the following edits:

> *Fig 1 and 2: some confusion and mixtures in text that needs to be looked at. Before describing figures A and B separately authors should explicit what orange blue and green plot correspond to in general, this can also be mentioned in the figure where they could add the ϵ parameter values next to each plot color. "AIC= value vs value", authors could explain for which eq vs eq each value corresponds. Titles of both these figures could be changed to more general description as this figure in my opinion shows more than tracking errors but is an example of PSD, Allan variance and comparison of fits.*
>
> *Fig 3: mark panels A, B, and C for consistency and easier description in legend for reader to better understand. Confusing histogram κ on bottom right which seems to show $\kappa = f(\kappa)$. Flip this graph 90 deg. to the right so it coincides with axis label of the 2D histogram. Add units to γ and κ in the axis labels.*
>
> *Fig 4: Description in legend difficult to follow, use lettering to describe the graphs. Describe better what are the plots in function of and explicit that corner frequency is f_c. Reorder sentences for better understanding of legend as well.*

We thank the reviewer for their feedback and have incorporated their suggestions. Specifically, we have added examples for the PSD and AV (without tracking errors) in the new introductory figure. We have adjusted the captions for Fig. 1 and 2. We have marked the panels, adjusted the caption, and added the units to the axes labels of Fig. 3. We have improved the legend and caption of Fig. 4.

> *With regard to the actual software After reviewal of the content of the program Tweezepy, I would suggest the following modifications:*
>
> > *Either creating an interface for such a program, which might be of interest for users who are not advanced in programming or python scripting and this might facilitate the general use of Tweezepy.*
> >
> > *Or to write more detailed documentation for users. The documentation I found ist not sufficient to make sure that any data obtained from instruments can be read in readily. Many of the parameters that need to be fed into the program are to my finding not so straightforward. As an example, the trace data has to be in nanometer per second for the MLE fit function to work (error message without an error description encountered otherwise). Explain this more detailed in the documentation what to feed into the various functions that the users might need for their calibration. Otherwise non-expert might be quickly uninterested and experts might not use the software because they have their own.*

We thank the reviewer for their feedback and have worked to improve the documentation. This documentation can be found online at `https://tweezepy.readthedocs.io` and included with the package in html and pdf formats. Additionally, the docstrings for the functions and classes, which include a description of the expected input and outputs (including units) can be accessed in Python by using the help() function. Furthermore, we will continue improving the user experience by handling bugs, documentation, and feature requests through the Tweezepy Github issues page (`https://github.com/ianlmorgan/tweezepy/issues`).

Reviewer two

The mathematical and analytical approaches are sound and the authors have done a good job in providing an overview of the critical steps in the process along with appropriate citations to the relevant literature. The examples are illustrative and the authors provide useful guidelines for improving the accuracy of the extracted parameters. I envision that this will be a useful tool for many single-molecule biophysicists who are interested in extracting the stiffness and drag coefficient of single-particle manipulation measurements.

We appreciate the reviewer's kind comments about the article and utility of the computational tool.

P 2, L 82-83: The statement regarding the insensitivity of thermal calibration approaches to the intrinsic parameters of the system is not strictly correct. This is correct for the simple variance based approach in equation 1, but not generally true since power spectral methods require accurate measures of the solution viscosity, the particle size, and the position of the particle relative to surfaces.

We note that the PSD method does not require an independent estimate of solution viscosity, particle size, or position of the particle relative to the surface. If only κ were estimated, then the reviewer would be correct, and these factors would need to be accounted for to estimate the fixed γ value; however, in our PSD method both γ and κ are independently estimated. Hence, these effects are implicitly accounted for in the resulting parameter estimates as has been previously shown for the position of the bead relative to surfaces in Ref. [1].

P5, L169-171: this sentence is confusing: "Conveniently this bias is negligible when τ_s/τ_s [37], so Eq. 9 can usually be applied, without further modification, to photodiode-based detection systems that include dead-time [44]" Typically one thinks of photodiode systems to be faster and exhibit no dead-time in comparison to camera based systems. Perhaps this could be better described based on the discrepancy between the effective bandwidth of the detector and the sampling rate – which leads to an effective dead-time perhaps. Without more details this statement seems incorrect and perhaps could be rephrased to get at the underlying issue.

In the text, we used photodiodes as an example of a detection system with dead-time; but, as the reviewer notes, these systems have subtleties that can be different from camera-based detection systems. However, this is not pertinent to our discussion, so we have modified the text (lines 185-187) to omit the discussion of photodiode systems. Instead, we clarify that Eq. 9 can be applied without modification, regardless of the detection system, if the sampling rate is higher than the corner frequency, i.e., $f_s > f_c$ and $\tau_s < \tau_c$.

P6 L182. Based on issues related to noise estimation and improving the robustness of the fitting, Flyvbjerg and coworkers, e.g. reference 11, have suggested binning the PSD results in a similar manner as the AV results. Can the authors comment on the choice to not bin the PSD in light of the suggestion that this improves fitting for optical trapping experiments?

We thank the reviewer for bringing this to our attention. Typically, the PSD is linearly binned, and we find that linearly binning provides accurate parameter estimates (Fig. 5). After rereading reference 11 (as well as reference 14 & 17), we believe that Flyvbjerg and coworkers perform Welch's method (using linearly bins) to calculate the PSD values first and then logarithmically bin them by frequency. This is different than what is done with the AV, which splits the trajectory into logarithmic bins before calculating the AV values. As noted in reference 17, the logarithmic binning of Flyberg and coworkers helps with visualization, but linear binning is recommended for parameter estimates using MLE. We have included a note in the text about this (lines 218-219).

Figure 3. The 2-D histogram has a mix of large "pixels" toward the center of the distribution but small point that lie outside the last contour line – can the authors explain these discrepant points?

The underlying plotting package uses a 2D histogram for dense packing of points within the contour lines and individual points for sparse packing of points outside the contour lines. We have updated the caption to clarify this.

For the data in figure 3 it would be helpful to compare this particular case to a Gaussian approximation. Or compare the uncertainties derived through the MC simulation process to those estimated from a Gaussian distribution.

We have updated the figure to include a Gaussian distribution overlay and the text (lines 403-404) to compare results from the two uncertainty methods.

> *Figure 4. It seems surprising that the bias in the stiffness is worse for the PSD model in which the drag coefficient is fixed at low cut-off frequencies. I would expect that the fit should improve, or be no worse, when the known drag coefficient is used in the fitting function. Can the authors comment on this somewhat surprising result?*

The reviewer appears to comment that fixing the drag coefficient causes the PSD model to become worse at low corner (cut-off) frequencies. This is not the case. At low corner frequencies, fixing the drag coefficient does not make a difference in the bias. The PSD method is more biased than the AV method at low corner frequencies because the linear binning reduces the frequency resolution. We suspect the confusion here was caused by our unclear method of plotting the points in the original submission; accordingly, we have changed the markers in the figure to improve visibility of all the situations, and so to clarify the differences between the methods.

> *The authors do not specifically address drift in the measurements. It would be useful to include a brief comment on how drift would manifest in the AV and PSD treatments of the data and how it could be included I the fitting or eliminated from consideration.*

We have included a short discussion of drift in the text (lines 388-395). Mechanical drift often manifests as $P(f) \sim f^{-1}$ at low frequencies in the PSD and $\sigma^2_{\mathrm{AV}} \sim \tau$ at long averaging times in the AV. Because the PSD generally has lower frequency resolution at low frequencies, we have found that drift is easier to identify and remove with the AV. In the AV, drift leads to an easily identifiable local minimum below which it does not significantly affect the measurements [4]. Hence, by limiting the fit to regions before the local minimum, accurate parameter estimates can be determined.

In addition to addressing the reviewer's comments, we have also made some small grammatical changes.

References

[1] Lansdorp BM, Saleh OA. Power Spectrum and Allan Variance Methods for Calibrating Single-Molecule Video-Tracking Instruments. Review of Scientific Instruments. 2012;83(2):025115. doi:10.1063/1.3687431.

[2] Cox DR, Snell EJ. A General Definition of Residuals. Journal of the Royal Statistical Society Series B (Methodological). 1968;30(2):248–275.

[3] Nørrelykke SF, Flyvbjerg H. Power Spectrum Analysis with Least-Squares Fitting: Amplitude Bias and Its Elimination, with Application to Optical Tweezers and Atomic Force Microscope Cantilevers. Review of Scientific Instruments. 2010;81(7):075103. doi:10.1063/1.3455217.

[4] Andersson M, Czerwinski F, Oddershede LB. Optimizing Active and Passive Calibration of Optical Tweezers. Journal of Optics. 2011;13(4):044020. doi:10.1088/2040-8978/13/4/044020.
