# Supplementary material for: Tweezepy: A Python package for calibrating forces in single-molecule video-tracking experiments
Source: PLoS One. 2021 Dec 31;16(12):e0262028. doi: 10.1371/journal.pone.0262028 (PMC8719779; doi:10.1371/journal.pone.0262028)
Supplement: Attachment — Submitted filename: tweezepy_second_replyv2.pdf [file pone.0262028.s002.pdf]

Reply to reviewer comments for Tweezepy

Ian L. Morgan, Omar A. Saleh

December 3, 2021

We thank the editor and reviewers for their effort and time. In particular, we appreciate reviewer one's feedback that they found the updated documentation to be helpful.

In response to the minor issues noted by reviewer one:

> *all axes should be labeled. for histograms, the y-axis is often not labeled (e.g. Fig 4). What about counts?*

We have labeled the histogram axes appropriately in Fig. 4 to indicate they represent counts.

> *for better referencing, I was missing in section 7 a refer to Fig. 1 - e.g. in lines 415/416. This makes it clear to the non-expert reader what are the simulated traces. In the same section, the authors can refer to Fig. 2 and 3 for the PSD and AV.*

We have adjusted the text in Section 7 to improve its clarity and included references to Fig. 1B on line 421 and Fig. 2B and Fig. 3B on line 425.

All changes have been marked in red.
